# Adaptive Gain Modulation in V1 Explains Contextual Modifications during Bisection Learning

**DOI:** 10.1371/journal.pcbi.1000617

**Published:** 2009-12-18

**Authors:** Roland Schäfer, Eleni Vasilaki, Walter Senn

**Affiliations:** 1 Department of Physiology, University of Bern, Bern, Switzerland; 2 Department of Computer Science, University of Sheffield, United Kingdom; University College London, United Kingdom

## Abstract

The neuronal processing of visual stimuli in primary visual cortex (V1) can be modified by perceptual training. Training in bisection discrimination, for instance, changes the contextual interactions in V1 elicited by parallel lines. Before training, two parallel lines inhibit their individual V1-responses. After bisection training, inhibition turns into non-symmetric excitation while performing the bisection task. Yet, the receptive field of the V1 neurons evaluated by a single line does not change during task performance. We present a model of recurrent processing in V1 where the neuronal gain can be modulated by a global attentional signal. Perceptual learning mainly consists in strengthening this attentional signal, leading to a more effective gain modulation. The model reproduces both the psychophysical results on bisection learning and the modified contextual interactions observed in V1 during task performance. It makes several predictions, for instance that imagery training should improve the performance, or that a slight stimulus wiggling can strongly affect the representation in V1 while performing the task. We conclude that strengthening a top-down induced gain increase can explain perceptual learning, and that this top-down signal can modify lateral interactions within V1, without significantly changing the classical receptive field of V1 neurons.

## Author Summary

Neuronal models of perceptual learning often focus on the feedforward information stream extending from the primary sensory area up to the prefrontal cortex. In these models, the stimulus representation in the sensory area remains unchanged during learning while higher cortical areas adapt the read out of the relevant stimulus information. An alternative view of perceptual learning is that the sensory representation at the very early cortical stage is modified by an adaptable top-down signal emerging from a higher cortical area. In this view, the sensory representation during perceptual training is sharpened by an internal signal which can be turned on or off depending on the task. We show how this top-down view explains improvements in interval discrimination by modifying contextual interactions in the primary visual cortex (V1). Without such top-down signal the V1 interactions support surface segmentation as it is typically used in visual scene analysis. During an interval discrimination task, however, a top-down signal which globally increases the gain of V1 neurons sharpens the V1 circuitry for this specific task. Although the top-down signal acts through a simple gain increase, the signal can change the tuning curves of V1 neurons embedded in a recurrent circuitry in a rather complex way.

## Introduction

Neurons in the primary visual cortex (V1) are driven by different sources. While early work emphasize the feedforward sensory input stream [1], later investigations highlight the strong recurrent connectivity within V1 [2] or the top-down modulation by higher cortical areas [3]. Recurrent and top-down connections are thought to mediate contextual interactions within V1 which shape the neuronal responses by additional stimuli in the non-classical receptive field. These contextual interactions are themselves modulated by perceptual tasks subjects perform [4]–[6]. It remains unclear, however, how the different input streams interact to generate the observed V1 activities, and how this supports perception and perceptual learning. Here we suggest a minimal connectivity model of V1 which integrates the bottom-up and top-down information stream in a recurrent network to support either surface segmentation [7],[8] or interval discrimination during a bisection task [6], [9]–[12].

**Figure 1:**
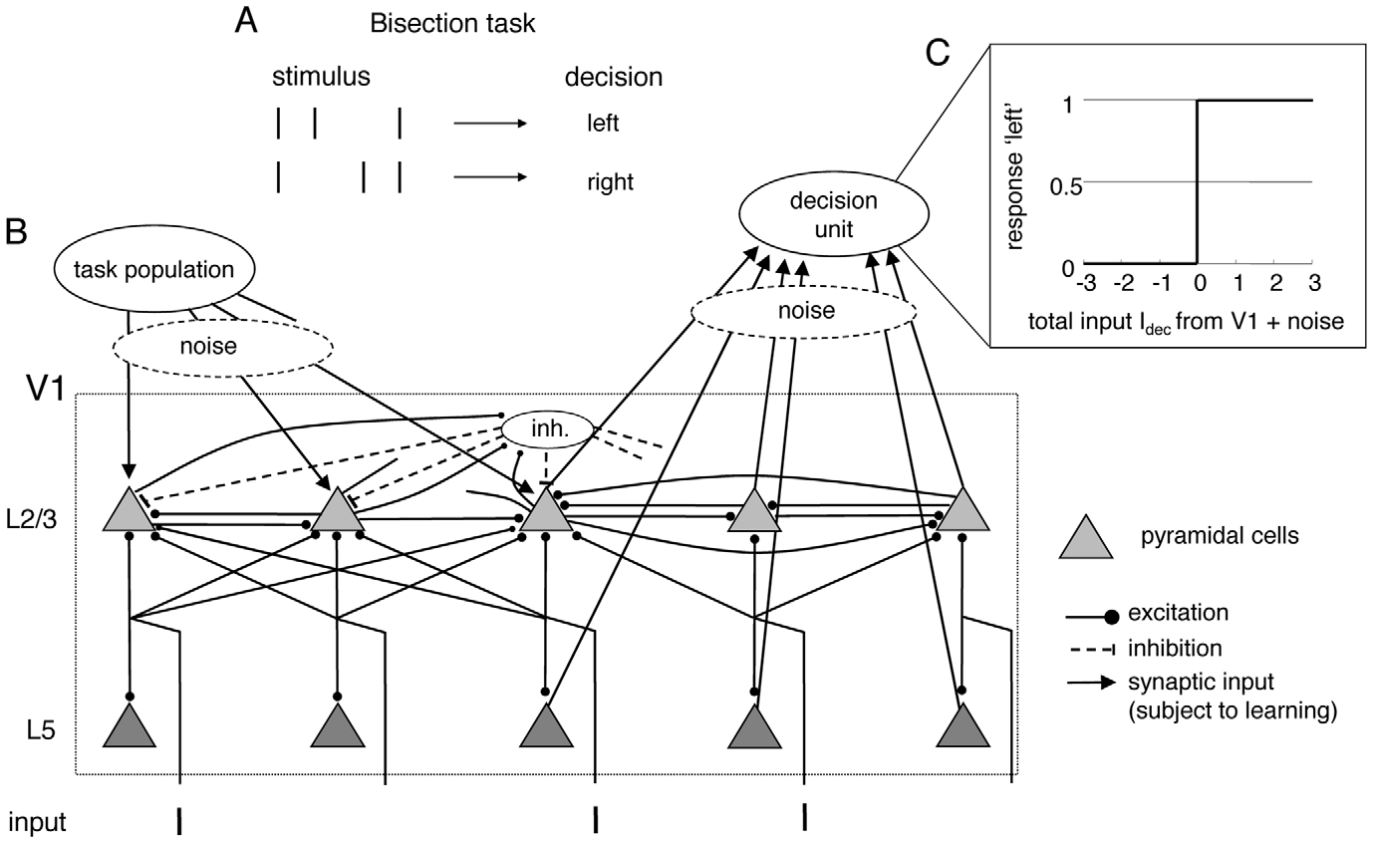
Bisection task and model network. (A) A bisection stimulus consists of three vertical lines, with the middle line slightly displaced from the center. The subject has to indicate whether this middle line is displaced toward the left or the right line. (B) Possible embedding of the model into a V1 circuitry. (Only a subset of the otherwise mirror-symmetric connectivity pattern in the V1-box is rendered, and to show the continuation, the initial segments of some connection lines are also drawn). Each of the bisection lines is activating (via non-modeled L4 neurons) a single L5 pyramidal neuron while projecting through a Gaussian fan out to the L2/3 pyramidal neurons. Both pyramidal layers project to a binary decision unit in a higher cortical area. The gain of the L2/3 pyramidal neurons is modulated by top-down input from a task population. L2/3 neurons are recurrently connected both through direct excitation and via a global inhibitory neuron. (C) The decision unit sums up and thresholds the weighted firing rates of the noisy pyramidal neurons. Learning consist in modifying these readout weights, as well as in a modification of the top-down input strength.

In the bisection task subjects are shown three small parallel lines and have to decide whether the middle line lies somewhat closer to the leftmost or to the rightmost line (Figure 1A). It has been suggested that the observed performance improvement in this perceptual task, due to repeated practicing, originates from a modulation of the sensory representation through long-term modifications of recurrent connections within V1 [13]. But it is difficult to reconcile this V1-intrinsic explanation of perceptual learning with the task-dependency of the modulation measured in the monkey V1 [6],[10]. In fact, the same two parallel lines as part of a bisection stimulus produced a completely different response behavior of V1 neurons depending on whether this stimulus was presented while the monkey was performing a fixation or a bisection task [10]. Moreover, these differences only occurred after an extended period of perceptual training. If the training-induced response modulations for bisection stimuli were explained purely V1-intrinsically, they would have been observed also for bisection stimuli alone, without performing the task. Hence, the experimental data is more readily explained if the modulations of the contextual interactions in V1 originate from a task-dependent top-down signal to V1 which is adapted during perceptual learning. Because the neuronal response curves evaluated at different line positions do not simply scale in a multiplicative way as a function of the task, a task-specific top-down mechanism was postulated which transiently modulates the strength of lateral connections and the excitatory/inhibitory balance within V1 [6],[10],[14]. Yet, how such a task-specific gating of lateral connections can be achieved remains elusive.

Here we show that – despite the complexity of the observed task-dependent modulation of the response curves – a simple top-down induced gain increase of V1 pyramidal neurons embedded in a recurrent circuitry can explain the various electrophysiological recordings including the psychophysics of bisection learning. Our model makes use of the same type of global gain increase in V1 neurons observed during attention [15]. As we show, combining the attentional signal with synaptic plasticity on the top-down connections to V1 leads to perceptual learning via a training induced strengthening of the gain increase. Even if the top-down signal acts globally (or at least semi-global within an area of the same hemisphere) on the pyramidal neurons, the intrinsic V1 wiring shapes this signal so that it leads to a strong and nonlinear response modulations for parallel lines in flanking positions, while only marginally affecting the response to a single line [10]. The top-down induced gain modulation puts the recurrent V1 network into different dynamical regimes. At low gain of the excitatory neurons, global inhibition uniformly suppresses responses to neighboring iso-orientation lines. At high gain, a winner-takes-all behavior develops so that global inhibition only suppresses weakly active neurons while competitively enhancing strongly active neurons. As a result we find that low gain supports surface segmentation through off-boundary suppression [7], while high gain supports interval discrimination through strengthened competition. Hence, training a (semi-)global attentional signal which modulates the gain of the excitatory neurons can shape the V1 circuitry to subserve different tasks.

## Results

### 

#### Model network

Our V1 model includes two layers of neurons, with the second being recurrently connected through excitation and global inhibition. Both layers project to a downstream readout unit which performs binary decisions based on the weighted input signals (Figure 1, B and C). To show how this model might be embedded into the architecture of V1 we tentatively assign the model neurons to cortical layers and neuron types, although such an assignment is not unique. In our choice, the visual input stream from the thalamus is transferred (via layer 4) to the pyramidal neurons in layer L2/3 and L5. The task-dependent neuronal modulation observed in the same hemisphere where bisection training occurred [10] is implemented by a top-down induced gain increase in L2/3 pyramidal neurons while performing the bisection task. Learning consists in modifying the readout weights from the noisy pyramidal neurons from both layers to the decision unit, and in increasing the strength of the global top-down signal (see Materials and Methods).

#### V1 nonlinearities and symmetry breaking

**Figure 2:**
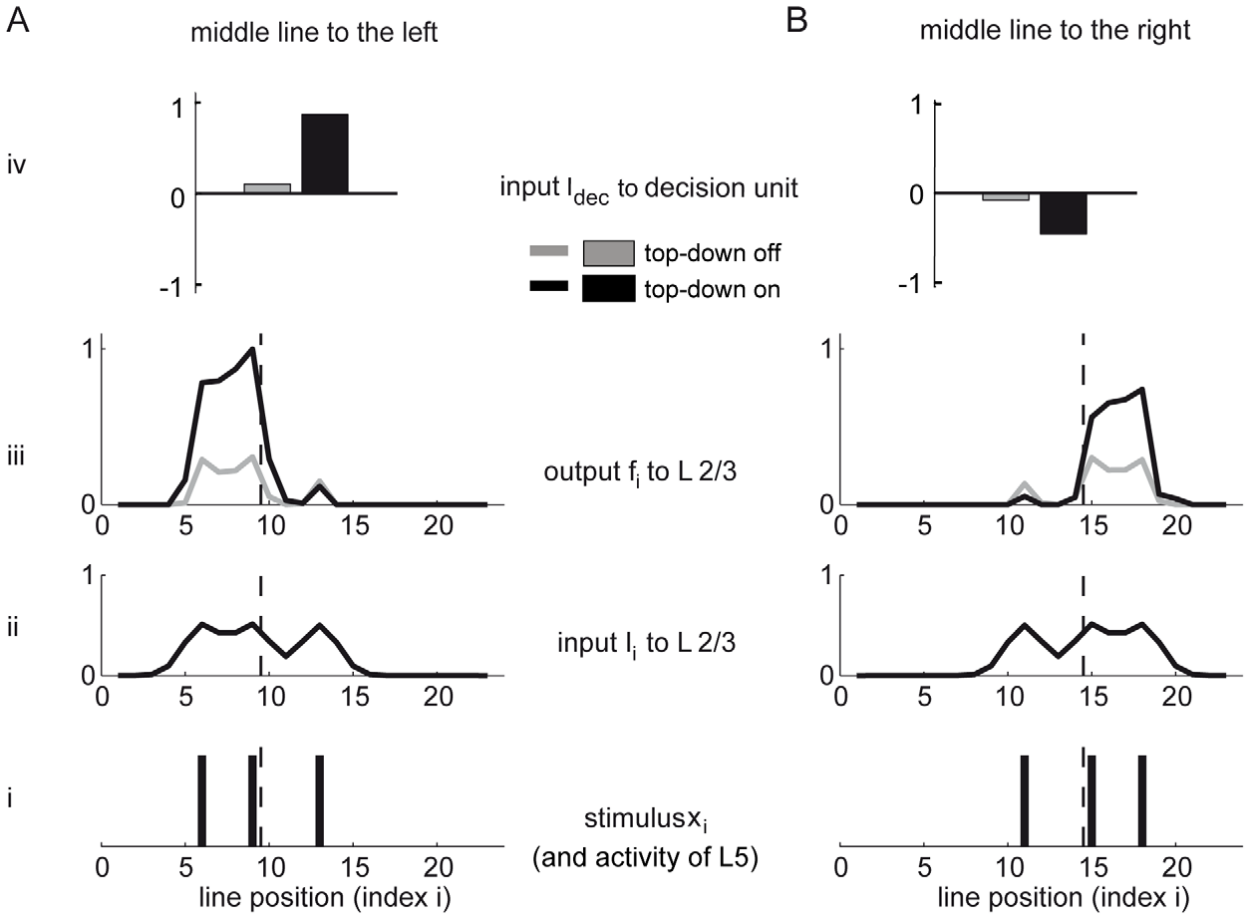
The top-down induced gain increase of the L2/3 neurons provokes symmetry breaking in the recurrent network and the resulting competition improves the signal-to-noise ratio underlying the bisection decisions. (A) and (B): Network activities for two mirror symmetric bisection stimuli after learning. (i) Each line in the bisection stimulus activates a neuron in L5 at the corresponding position (dashed line indicates the stimulus center). (ii) The feedforward input to the L2/3 pyramidal neurons is locally spread. (iii) Local recurrent excitation and global inhibition competitively suppresses L2/3 pyramidal neurons receiving weak input, leading to a lateralization of the activity to the side of the middle line (A: left; B: right). An additional top-down gain increase enhances this lateralization (black versus grey lines). Deviations from mirror symmetry in the responses are due to a stochastic modulation of the lateral connectivity in L2/3. (iv) The input to the decision unit, 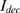, is a weighted sum of the noisy L2/3 and L5 activities without (grey) and with (black) top-down input, upon which the decision ‘left’ or ‘right’ is made by thresholding at 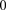. The weak gain increase (by a factor of 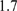) dramatically increases the signal (by a factor of 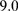 and 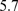, respectively). The plots show averaged activities over 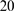 runs with the same stimulus configurations.

The task of our model network during successful bisection discrimination is to deliver a supra-threshold input current from the pyramidal neurons to the decision unit (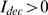) if, say, the middle line of the bisection stimulus is shifted towards the left. Analogously, it should deliver a subthreshold current (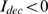) as soon as the middle is slightly shifted towards the right of the interval center (Figure 2, a and d). For simplicity we assume that the decision threshold is 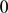 and that the readout weights can be positive or negative. If the stimulus representation in V1 is to support the decision process, the V1 activity should nonlinearly change when the middle line moves across the bisection center. A suitable activity switch is achieved by a positive feedback among the L2/3 pyramidal neurons and their competition via global inhibition. The recurrence provokes a symmetry breaking in the L2/3 activity and further lateralizes any slight deviation of the activity to either side from the bisection center (Figure 2, b and c). This arises because a locally dominating cluster of L2/3 activity suppresses weaker input via inhibitory feedback. Thus, if the middle line is closer to the leftmost line, for instance, this suppresses the L2/3 representation of the right line but enhances the representation of the two proximal (middle and left) lines. A top-down induced gain increase of the L2/3 pyramidal neurons further enhances the positive feedback and the competition, thereby improving the signal-to-noise ratio (Figure 2, c and d; for a thorough analysis of the L2/3 dynamics see Materials and Methods).

**Figure 3:**
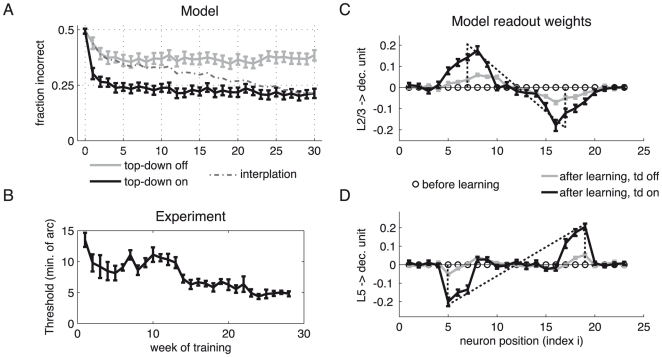
Performance and evolution of the readout weights during bisection training. (A) Fraction of erroneous network decisions against training week, with a ‘week’ consisting of the presentation of 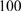 bisection stimuli of fixed outer-line-distance (‘width’) but with randomized positions. Upon each stimulus presentation, the readout weights from the L5 and the L2/3 pyramidal neurons to the decision unit were changed according to an error correcting learning rule. A top-down induced gain increase in the L2/3 pyramidal neurons reduces the error level (grey: gain factor 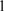; black: gain factor 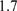). Hence, a substantial improvement in performance is achieved if learning simultaneously increases the top-down input strength, leading to a learning curve which interpolates between the two curves (dashed line). The fast initial learning progress arises from adapting the readout connections to the decision unit. (B) Learning curve for a monkey performing the bisection task (adapted from [10]). (C) Synaptic weights from L2/3 pyramidal neurons to the decision unit before (circles) and after learning with (black) and without gain increase (grey). The dotted line indicates the universal weight distribution inferred in the theoretical argument. (D) Same as in C, but for synaptic weights from L5 pyramidal neurons to the decision unit. Error bars represent standard error of the mean (using 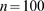 learning runs).

If the bisection center is always located in the same place, these nonlinear interactions within the L2/3 layer enable a downstream readout unit to robustly discriminate between left and right interval bisections, independently of the bisection width (for instance by assigning positive readout weights to the left, and negative readout weights to the right pyramidal neurons, see Figure 3C). However, because we require that the task be solved for different positions of the bisection stimulus, the absolute position of the L2/3 activity must be re-expressed as a relative position with respect to the flanking lines of the bisection stimulus. This is achieved by comparing, and in fact by subtracting, the activity in L5 from the one in L2/3 (as expressed by the readout weights to the decision unit, Figure 3C and D).

#### Bisection learning and signal-to-noise ratio

The initial performance increase in the bisection task is achieved by modifying the readout weights from the L5 and L2/3 pyramidal neurons to the decision unit (Figure 3). The weights are adapted according to the perceptron rule, an error correcting learning rule which can be interpreted as an anti-Hebbian modification of the synaptic strengths in the case of an error (see Materials and Methods). The early saturation of the learning curve is caused by the limited signal-to-noise ratio imposed by the network. Without stochasticity in the pyramidal cell output, the errors would vanish. Because the noise is additively incorporated in the pyramidal cell firing rates, however, some errors remain as in the experimental data.

Learning of the readout weights cannot further improve the signal-to-noise ratio. In fact, rescaling the readout weights would equally rescale the signal and the noise. However, if the gain of the L2/3 pyramidal neurons is increased by top-down input, the signal is amplified prior to the addition of noise, and this does improve the signal-to-noise ratio. As a consequence, the performance also improves and the learning curve decays to a lower error value (Figure 3A). The gain increase by a factor of 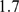 - which is comparable in size with the dendritic gain modulation observed for pyramidal neurons *in vitro* [16] -reduces the asymptotic error level by roughly one half. The gain may also adaptively increase throughout the learning process by modifying the top-down connection strength. While learning the readout connections improves the performance in an initial phase starting at chance level, learning the top-down connections contributes to the main improvements later on (Figure 3A). We therefore hypothesize that the learning effect in monkeys (Figure 3B) similarly originates mainly in enhancing the task-induced gain increase in V1 pyramidal neurons.

#### Network readout: a theoretical consideration

To further understand how the network masters the bisection task for varying bisection positions we first formalize the problem in simple algebra. Let us assume that the left, middle and right line of the bisection stimulus are at positions 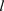, 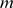 and 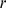, respectively. The condition that the middle line is more to the left (i.e. that the right interval is larger) is then expressed by 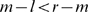, or 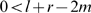.

To turn this algebra into a network calculation we set the readout weight of the L5 pyramidal neuron at position 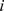 to 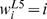. If the activities 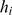 of the L5 pyramidal neurons at position 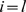, 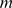 and 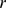 are 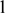 and those of the others 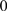, the decision unit will receive the input current 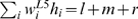 from L5. We now set the readout weight of the L2/3 pyramidal neurons to 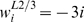 and assume that the recurrent excitation with the global competition in L2/3 implements a pure winner-takes-all dynamics. Because the pyramidal cell at the position of the middle line 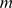 receives the strongest input via the two neighboring line stimuli left and right, the activity at this middle position will dominate (say with value 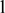) while the activity at the flanking positions will be fully suppressed. This leads to a current of 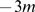 from L2/3, and together with the L5 input the decision unit receives the total input current 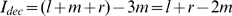. But since according to the above algebra the middle line is more to the left if and only if 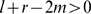, thresholding 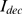 at 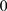 yields to the correct decision in the bisection task for all 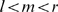, and hence for all bisection positions and bisection widths (for a more general consideration see Materials and Methods).

The above reasoning is confirmed by the simulations in which the readout weights adapt during the learning procedure such that after learning they roughly follow the theoretically calculated linear ramps (see Figure 3, C and D). Note that any vertical shift of the L5 readout weights by some constant offset, 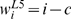, can be compensated by an appropriate offset in the readout weights from L2/3, and that an additional common factor in front of the weights can be absorbed in the corresponding presynaptic neuronal activities.

#### Task-dependent modulation of V1 interactions

**Figure 4:**
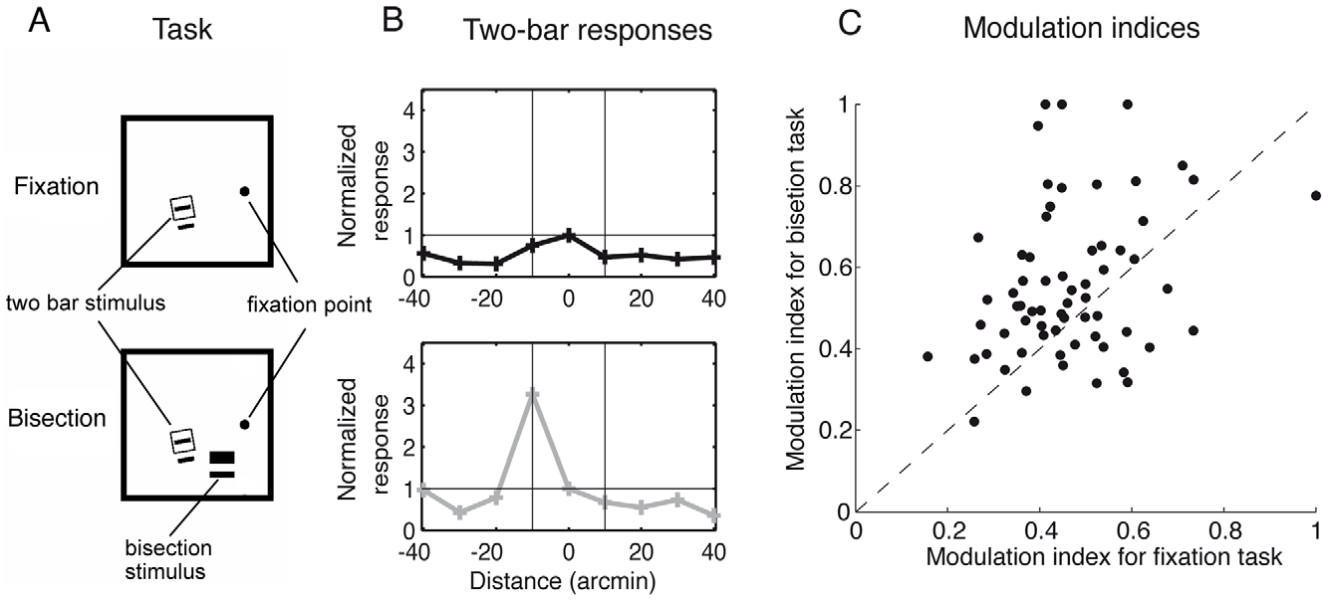
Learning-induced gain modulation in L2/3 pyramidal neurons qualitatively changes the local interactions. (A) In the experiment [10], monkeys were performing either a fixation task (top) or a bisection task (bottom) while the activity of a supra-granular V1 neuron was recorded in response to a two line stimulus in a side-by-side configuration. One of the two lines is centered in the receptive field (sketched by the square) of the recorded neuron. (B) Activity of the corresponding model L2/3 pyramidal neuron mimicking the recorded supra-granular neuron for different positions of the flanking line, with individual curves normalized by the activity with flanking line at 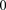. Top: During pure fixation or before training (modeled by a non-modulated circuitry, gain 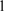), the response of the central neuron is suppressed by the flanking line via global inhibition. Bottom: When performing the bisection task at a nearby location in the trained hemisphere (modeled by a top-down induced gain increase of the L2/3 pyramidal neurons from 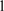 to 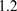) the lateral suppression turns into strong excitation at random positions due to the enhanced competition within the stochastically modulated network. (C) Modulation indices for the ‘bisection task’ (gain 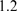) versus ‘fixation task’ (gain 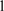). The *modulation index* is defined as the normalized difference between the maximal and minimal response of the recorded L2/3 pyramidal neuron, each evaluated for the different positions of the flanking line (as represented in B, see Materials and Methods). Evaluation for neurons in 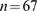 stochastic network configurations shows that the modulation index under the bisection condition is significantly larger than under the fixation condition (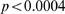 for paired t-test with 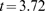), as it is also observed in the experiment ([10] with 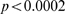, 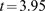, 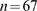).

While the theoretical calculation assumes a winner-takes-all mechanism, a smoothed version with a winner-takes-most provides enough nonlinearity to allow for correct bisection decisions independently of the stimulus position and width. In the model, the winner-takes-most behavior emerges from the local-excitation global-inhibition network in L2/3 by a global gain increase in the L2/3 pyramidal neurons. To visualize this transition we monitor the activity of a L2/3 pyramidal neuron driven by a line in its receptive field while changing the position of a second flanking line. At low neuronal gain - mimicking the performance in the fixation task, or the performance in the untrained hemisphere for the bisection task - we observe the classical lateral inhibition of the response by the flanking line (Figure 4B, upper row), in accordance with experimental findings [10]. If the gain factor is increased (from 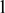 to 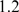) - mimicking the effect of training and subsequently performing the bisection task at some nearby position - the lateral inhibition turns into excitation at some of the positions (Figure 4B, lower row), as has also been observed in the experiment.

The switch from inhibition to the randomized excitation pattern occurs because the high gain strengthens the positive feedback among L2/3 pyramidal neurons while the recurrent inhibition cannot counterbalance the strengthened excitation (since the gain of the inhibitory transfer function at high inputs is lower, see Materials and Methods). Because of the stochastic modulation of the otherwise symmetric recurrent connectivity between each pair of L2/3 pyramidal neurons, there is a 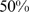 chance that one of two pyramidal neurons, each driven by its own line stimulus, wins over the other. Hence, when stimulating with two lines and recording from a model pyramidal neuron with one of the lines in its receptive field center, the activity may either be enhanced or suppressed while presenting a second flanking line (Figure 4B, lower row). The statistical evaluation of the modulation indices for the L2/3 model pyramidal neurons when changing from the fixation to the bisection task roughly matches the experimentally extracted modulation indices (see Figure 4C and [10]).

#### Largely task-independent receptive field properties

**Figure 5:**
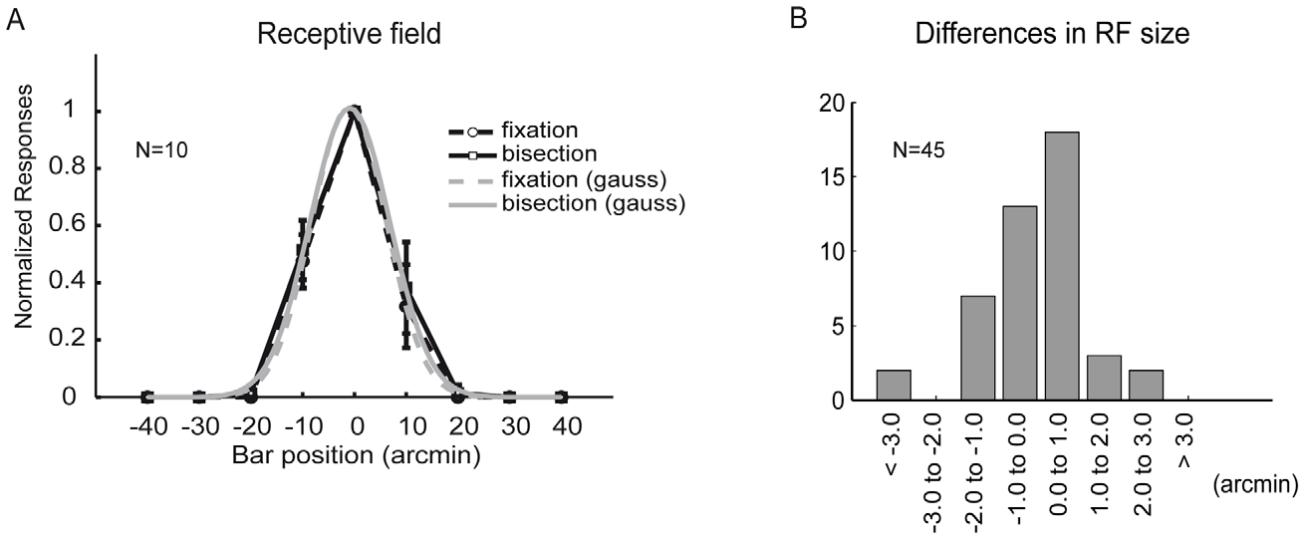
Largely task-independent receptive field of L2/3 neurons. (A) Averaged normalized responses of one typical L2/3 model pyramidal neuron to a single line placed at different positions for the un-modulated network (‘fixation task’, gain 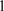, dashed line) and with a top-down induced gain increase of the L2/3 pyramidal neurons (‘bisection task’, gain 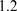, solid line). Grey lines show Gaussian fits (with 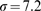 and 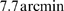 for the fixation and the bisection task, respectively). Error bars arise from the stochasticity in the top-down induced gain modulation (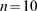 line presentations at each position with fixed network configuration). (B) Histogram of the differences in the receptive field (RF) size of 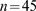 model pyramidal neurons under bisection versus fixation conditions. For comparison with the experiment where the same number of neurons were recorded from different positions and animals, we extracted the model neurons from 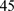 different network configurations and determined the receptive field as in A. The difference in the receptive field size was not significant (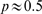 in the t-test with 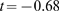), in agreement with the experimental findings ([10], with 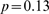, 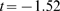, 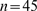). However, increasing the number of sample neurons may turn a non-significant into a significant result, and for the model this is in fact the case, with RF size during the bisection task becoming significantly (in terms of the t-test) smaller by 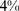 than without performing this task.

Despite the strong task-induced response modulation for the two-line stimuli after training (up to a factor of 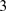, compare Figure 4B top and bottom), only a very minor modulation of the receptive field of the L2/3 pyramidal neurons is observed in the model. The receptive field was determined by the average response to a single line stimulus presented at the different positions, once with gain 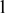 (mimicking the performance of the fixation task or the performance of the bisection task before training), and once with a gain of 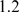 of the L2/3 pyramidal neurons (mimicking the performance of the bisection task after bisection training in the same hemisphere, see Figure 5A). On average, the gain increase leads to only a slight reduction of just 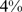 in receptive field size. This is in line with experimental findings on task-induced changes in the receptive field for single-line stimuli which failed to be statistically significant [10]. This also happens in our model if we estimate receptive field sizes based on the same number of measurement as in the experiment (Figure 5).

The strong modulation effect for the two- and three-line stimuli arises because these multiple line stimuli nonlinearly recruit additional parts of the recurrent L2/3 pyramidal cell circuitry. In contrast, the one-line stimulus used to sample the receptive field is too weak to recruit the recurrent network.

#### Learning transfers to other bisection widths

**Figure 6:**
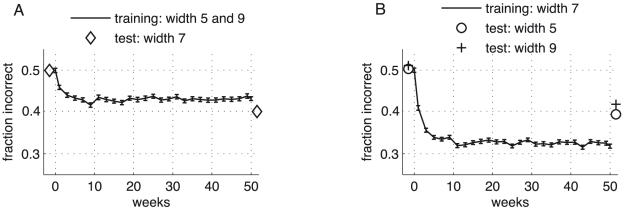
Training under stimulus roving and transfer to untrained stimulus widths. (A) Fraction of incorrect network decisions for the combined training with two stimulus widths (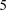 and 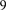) which were randomly interleaved (‘roving’). In agreement with recent findings [12] – but unlike previous predictions [11],[13] – learning under stimulus roving is impaired, although still possible (the final fraction of incorrect responses, being 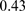 for stimulus roving, is reduced for individual training of the bisection width 5 and 9 to 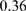 and 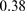, respectively). Note that the post-training test shows a better performance for the interpolated width 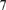 which was itself not trained. (B) Learning curve for bisection stimuli of width 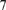 (line), with pre- and post-learning tests for the untrained stimulus widths 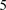 and 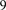. A learning transfer of roughly 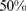 from the trained to the two untrained widths is predicted by the model. Error bars represent the standard error of the mean evaluated for 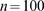 runs.

Our model is also compatible with the recent psychophysical finding [12] showing that some performance increase is still possible under stimulus roving, i.e. random permutation of two bisection widths during training (Figure 6A), although the learning is impaired both in the data and the model. The model moreover predicts that the learning progress transfers to an untrained bisection width, provided that the untrained width is in between the two widths of the trained bisection stimuli.

Interestingly, the performance on the untrained interpolated width is slightly better than the performance on the trained neighboring widths (Figure 6A). This can be explained by the fact that training with two bisection widths forces the readout weights to move closer to the universal linear ramp calculated above (Section “Network readout: a theoretical consideration”, data not shown) and makes the bisection discrimination more robust for the interpolated width. Training with a single bisection width, in turn, leads to an improved performance for this specific width, but the transfer of learning to a non-trained width is impaired (Figure 6B). Nevertheless, the simulations confirm the experimental observation that some learning transfer is possible when increasing or decreasing the stimulus width by roughly 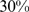 [11].

## Discussion

We have shown that a considerable part of the improvement in bisection learning can be explained by the adaption of an attention-like, global top-down signal to V1. This explanation shifts the view of perceptual learning from being stimulus driven to being attention driven. Since our model network is initialized with random readout connections without assuming any prior knowledge about the task, the top-down mediated performance increase is preceded by an initial phase of also adapting the readout connections from V1 to a decision unit. The key assumption of our model is that ‘perceptual attention’ increases the gain of sensory neurons, in our interpretation of recurrently connected L2/3 pyramidal neurons in V1. This gain increase strengthens the competition within the V1 circuitry and nonlinearly shapes the stimulus representation to improve the readout by the decision unit. The model explains the experimental observation that during the bisection task the interaction between V1 neurons representing two parallel lines changes from mutual inhibition to a randomized excitation-inhibition pattern, without significantly changing the classical receptive field properties of the involved neurons [10].

### 

#### Other models and explanations

Our approach of explaining perceptual learning by adapting a top-down signal has to be contrasted to the dominant view of perceptual learning as an adaptation of the readout connections only, or of a long-term modification of the sensory representation within V1 ([13], for a review see [17]). Apart from an early model for Vernier discrimination [18] and a recent model for brightness discrimination [19], surprisingly little computational studies on top-down effects in perceptual learning exist, despite abundant experimental evidence [5],[6],[9],[10],[14]. This may be related to the fact that, as in the present case, the phenomenology of the task-dependent modulation of the contextual interactions is quite rich, and at a first glance, would require elaborate top-down gating of recurrent connections in V1 going beyond attention, as suggested in [6],[10],[14]. However, as our model shows, a simple (semi-) global attentional signal which modulates the gain of pyramidal neurons [15],[16] may produce non-multiplicative modulations of the response functions within a recurrent V1 circuitry when sampled by two parallel lines, or no significant modulation when sampled by one line only (see Figures 4B and 5, respectively). Hence, a multiplicative gain modulation underlying perceptual learning can be masked by the distorting recurrent processing, or be overlooked by its small effect on the classical receptive field.

#### Generality of the network architecture

The proposed implementation could itself be part of a wider V1 circuitry including neurons selective to different orientations [20],[21] or motion directions [22],[23], or part of a circuitry explaining contrast modulation [24],[25] or extra-classical receptive fields [26]. While adaptable top-down connections have been identified to project from higher visual areas to the supragranular layers of V1 [27],[28], they have also been shown to modulate the gain of pyramidal neurons in the sensory cortex [15],[16],[29]. The global inhibition among L2/3 pyramidal neurons assumed in our model could be mediated by a population of electrically coupled inhibitory neurons in the supragranular layers [30]. We have shown that these ingredients are sufficient to explain various task-dependent and learning-induced modifications of contextual interactions in V1.

The mechanism of a top-down induced gain modulation may represent a universal building block for cortical computation which extends beyond the specific example of bisection discrimination. Another example of perceptual learning which can make use of the same top-down interaction is the brightness discrimination task [5]. Here, a top-down induced gain increase – together with a top-down drive of inhibition – was shown to suppress the distorting interaction in V1 induced by collinear flankers, and this in turn explains the improvement in brightness discrimination [19]. An adaptive gain modulation which enables global competition has more generally been recognized as a versatile computational element in higher cortical information processing such as in spatial coordinate transforms [31], analog-digital switches [32], or in determining the granularity of category representations [33]. Along the visual pathway, a hierarchy of maximum-like operations was shown to be a universal non-linearity which enables position invariant object recognition [34]. Such maximum operations could be implemented in a task-dependent manner through our top-down modulated micro-circuitry which determines the position of the maximum by the recurrent L2/3 network and reads out the value of this maximum from the unperturbed L5 activity.

#### Experimental predictions

Our model is also consistent with recent psychophysical observations on bisection learning. In contrast to the alternative model that perceptual learning is based on modifying intrinsic V1 connections [13], it confirms improvements in bisection learning under stimulus roving [12], and a weak learning transfer from a trained to a non-trained stimulus width [11]. It moreover makes several testable predictions both on the behavioral and the neuronal level:

1. It further predicts a full learning transfer from two simultaneously trained, narrow and wide bisection widths to an untrained width lying in between the two (Figure 6A).
2. Since the feedforward and recurrent projection widths within V1 set an intrinsic scale for which symmetry breaking in the stimulus representation is strongest, bisection learning is predicted to deteriorate if the width of the bisection stimuli extends beyond this scale, say beyond 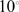 (cf. [11] for such a tendency). On the other hand, the performance of the bisection learning is predicted to be recovered if the bisection lines get themselves proportionally wider with the stimulus width (see Materials and Methods for more details).
3. The emphasis on the attention induced perceptual improvements predicts that perceptual learning could actually be achieved by a task-specific attentional training alone which would enhance the top-down induced gain increase in V1. Recent psychophysical results in fact suggest that pure mental imagery without presenting the full bisection stimuli can lead to improvements when subsequently testing bisection discrimination with real stimuli [35].
4. On a neuronal level, the model finally predicts that the V1-representation of a bisection stimulus during task performance switches when the right bisection interval becomes wider than the left (compare Figure 2B with 2A). This switch can be experimentally tested by recording from a V1 neuron with a receptive field in between the left and the middle line of the bisection stimulus. Assume that the right subinterval is initially smaller than the left one (but that the rightmost line nevertheless lies outside of the neuron's receptive field). While recording from the neuron, the rightmost line can be moved even further to the right, so that eventually the right subinterval becomes larger than the left one. At this point a nonlinear increase in the activity recorded from the neuron will be observed according to our model. Note that the predicted activity increase would represent a paradoxical extra-classical receptive field effect since the neuronal response becomes stronger when a line outside the classical receptive field is moved even further away. Such an experiment would provide strong evidence that perceptual training leads to a task-induced gain increase which produces competitive neuronal interactions within V1.

## Materials and Methods

### Model description

#### Network architecture

We consider linear arrays of 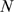 (in our simulations 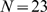) pyramidal neurons in L5 and L2/3 and an additional neuron in L2/3 representing the inhibitory population (see Figure 1). The 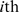 afferent projects to the 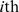 L5 neuron, generating a 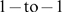 copy of the input 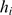 (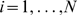) in L5. The afferents further project with a Gaussian fan out to L2/3, with synaptic connection strengths
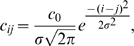
(1)from the 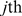 afferent to the 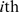 L2/3 pyramidal neuron (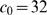, 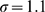). Since the comparison with the experimental data only involves normalized neuronal activities, we use arbitrary units in specifying weights and activities. Assuming that the visual stimuli for two adjacent V1 afferents are separated by an angle of 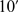 in the visual field, the width 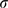 of the feedforward projections corresponds to a visual angle of 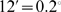.

The strength 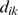 of the recurrent excitatory connection from the 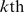 to the 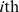 L2/3 pyramidal neuron (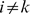) is
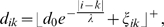
(2)with 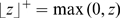, 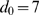, recurrent projection width 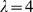, and 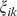 being a Gaussian random variable sampled from a distribution with mean 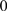 and standard deviation of 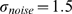 and cut-off at 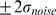. The strength of the self-recurrent weights is 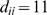. Note that the decay constant 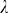 of the recurrent connections corresponds to a visual angle of 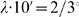.

#### Dynamics of L2/3 pyramidal neurons

The firing rate 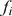 of a L2/3 pyramidal neuron receiving a total input current 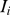 obeys the differential equation
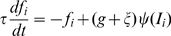
(3)with a time constant 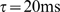. 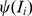 is the steady state transfer function defined as
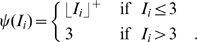
(4)


The factor 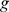 in (3) represents the neuronal gain which is modulated by a (not modeled) top-down signal. During the fixation task we set 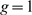, during the bisection processing at a nearby location 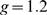 (Figure 4 B, lower row, and Figure 5), and during the direct involvement in bisection processing 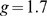 (Figure 2, 3, 6). To mimick the variability in the top-down input strength, Gaussian noise 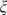 was added to the gain factor. For each stimulus presentation 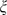 was drawn anew from a Gaussian distribution with mean 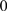, standard deviation 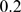, and cut-off at 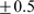.

The total input current to the L2/3 pyramidal neurons is composed of the current from the feed-forward afferents mediating the stimulus, and the recurrent excitatory and inhibitory connections within L2/3, respectively,
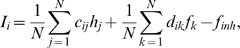
(5)with connection strengths 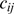 and 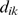 given in Eqs 1 and 2. An example of inputs and outputs of the L2/3 pyramidal neurons is shown in Figure 2.

#### Dynamics of the inhibitory L2/3 neuron

The firing rate of the inhibitory neuron appearing in Eq. 5 follows the differential equation
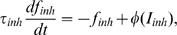
(6)with a time constant 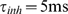 and a piece-wise linear steady-state transfer function
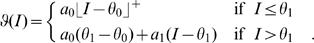
(7)The thresholds of the transfer function are set to 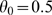 and 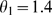, and the gain parameters are set to 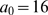 and 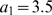. The total input current to the inhibitory neuron in Eq. 6 consists of the recurrent input from the L2/3 pyramidal neurons,
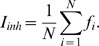



#### Stimulation protocols and numerical methods

In the bisection task, three afferents to V1 were stimulated corresponding to the left, middle and right line of the bisection stimulus (generating in L5 the activities 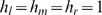 while the others L5 activities remained 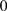). The width of the bisection stimuli, 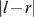, was 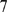 (Figure 3), and 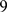 and 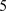 units (Figure 6), respectively. The delimiting lines (constrained to define a certain stimulus width) and the middle line were randomly and uniformly varied.

For Figure 3, bisection stimuli of width 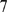 were presented, with middle line at one of the central 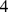 positions within the bisection stimulus, and delimiting lines varying from absolute position 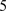 to 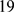 within the 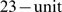 input layer. For Figure 6 we used stimuli with outer delimiting lines varying between positions 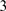 and 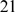 of the 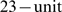 input layer, while the relative position of the middle line within the bisection stimulus varied across the central 4 stimulus positions. The statistics are based on 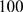 learning runs across 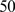 ‘weeks’. Before and after a learning run, performance tests on the non-trained stimulus width(s) were performed consisting of 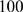 stimulus presentations each. For Figure 4, B and C, only two, and for Figure 5 only one afferent into V1 was stimulated, i.e. clamped at activity 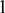 during the whole trial.

In all simulations a stimulus presentation (‘trial’) lasted for 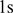, and in this time the recurrent network was relaxed to a steady state. Numerical integration was performed with the Runge-Kutta-Fehlberg-(4,5)-method of the Gnu Scientific Library which includes an automatic step-size control. Initial activities of all neurons were set to zero.

#### Decision making

The readout unit in a higher cortical area receives inputs from the V1 pyramidal neurons in L5 and L2/3. In the bisection task, the readout unit makes a binary decision about the position of the middle line depending on the weighted sum of the pyramidal neuron activities which are perturbed by additive Gaussian noise. The total postsynaptic current of the decision unit is given by
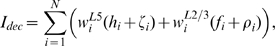
(8)where 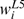 and 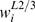 represent the input strengths from the 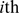 L5 and 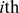 L2/3 pyramidal neuron to the decision unit, respectively (see Figure 1). The 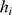
's represents the activity in L5 which is a copy of the stimulus, the 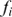
's are given by Eq. 3, and 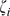, 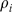 are independent Gaussian random variable with mean 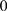 and standard deviation 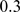 and cut-off at 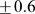. The binary decision 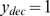 is taken if 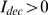, and 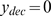 if 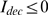. This decision is interpreted as the middle line being displaced to the left and right, respectively (cf. Figure 1C). An example of calculating 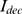 based on the pyramidal neuron activities and the different top-down inputs is shown in Figure 2.

Note that our model assumes different noise sources. The noise in the recurrent weights (Eq. 2) and in the gain factor 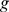 (Eq. 3) are both required to endow the activity distribution and the modulation index with a realistic degree of jitter as observed in the data (cf. Figure 4B and C, respectively). The additive noise in the L2/3 readout neurons makes the top-down induced gain increase a necessary ingredient in improving the signal-to-noise ratio by selectively enhancing 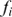 but not 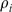. The fact that this noise is multiplied by the readout weights in Eq. 8, on the other hand, prevents an improvement of the signal-to-noise ratio by only up-scaling the readout weights.

#### Perceptual learning

Perceptual learning during the bisection task was implemented by an error-correcting plasticity rule for the synaptic weights 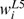 and 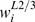 projecting from the pyramidal neurons to the decision unit. If the network decision was correct, no synaptic changes occurred. However, if the network decision was incorrect, the synaptic weights were updated in a anti-Hebbian way according to
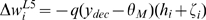


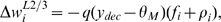
(9)with a learning rate 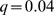 and a modification threshold 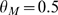. To avoid introducing an additional inhibitory neuron, we allow the weights to take on positive or negative values. An example of the synaptic strengths 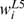 and 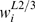 before and after learning is shown in Figure 3, C and D.

#### Modulation index

To quantify the modulation of the single neuron response by a flanking line during the different perceptual tasks we extracted the modulation index as in [10]. In the model, a fixed reference line activates the central input neuron at position 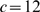, and a simultaneously presented flanking line activates one of the remaining input neurons 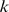 (with 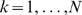; 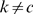, 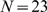). After network relaxation the response 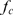 of the central L2/3 pyramidal neuron is extracted (with index fixed to 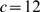), and the maximal and minimal response of 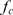 for the 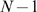 flanking line positions 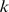, 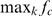 and 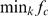, is determined (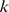 as above). The modulation index 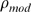 is then calculated by
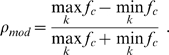
(10)This modulation index is calculated once with a gain factor 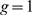, mimicking the simultaneously performed fixation task, and once with 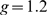, mimicking the nearby performance of the bisection task (Figure 4C). Figure 4B shows an example of the activities 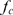 for different positions 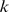 of the flanking line in the case of the fixation task (top) and the bisection task (bottom). The jitter in the simulations arises from the stochasticity in the connections among the L2/3 pyramidal neurons, 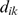 (see Eq. 2).

### Mathematical analysis

#### A one-layer perceptron is not enough

We first note that no neuronal readout from a single 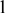-dimensional spatial layer exists which assigns any triplet of positions 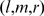 to one of two classes, depending on whether 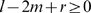 or 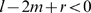. Note, however, that according to these inequalities, the problem is linearly separable when considering as an input space the 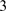-dimensional space of position triplets – instead of the 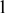-dimensional array of binary neurons. The non-existence of the 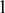-dimensional neuronal readout follows from a more general theorem, originally proved by Minsky 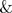 Papert, according to which no perceptron can translation invariantly detect local features [36, Theorem 10.4.1].

To reproduce the argument in the current setting we consider a perceptron defined by the weight function 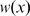 which classifies the stimuli 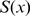 by 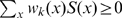 or 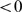, depending on whether 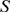 belongs to class 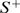 or 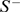, respectively. To keep with the intuition of discrete weights, the position variable 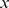 here is considered to be an integer. Translation invariant classification of stimulus 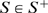 implies that 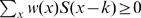 for all integers 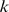, or equivalently, 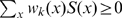 for all 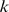, where 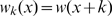. By linearity, convex combinations of two solution functions are again solutions, and in particular also 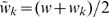 for any 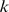. Iteratively averaging 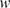 with the translates of the averages yields a progressive smoothing of the original weight function which converges to 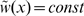. But a perceptron with constant weights can only distinguish stimuli based on their summed strength, 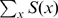. In particular, it cannot tell whether the widths of the stimuli in a class are bounded, or whether the stimuli extend across arbitrary long segments. Hence, if we require translation invariant classification, the stimuli in a class cannot be characterized by a local feature (as, e.g., it would be the case for bisection stimuli).

#### Condition on the nonlinear network interactions

As shown by the theoretical consideration in the Results, introducing a second layer in which the activities from the outer lines are fully suppressed while the activity from the middle line is unaffected, solves the problem. Possible weight functions defining the readout from the first (L5) and second (L2/3) layer are 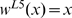 and 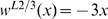 (see Results). Denoting the activity distribution across L2/3 by 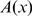, the input from this layer to the perceptron becomes 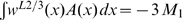, with 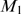 defining the *first order moment of* 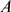, i.e. 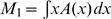 (to simplify the analysis below, 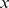 is a 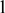-dimensional continuous variable). Note that for a normalized activity integral, 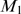 corresponds to the center of gravity of 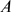.

In the case that 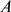 is a delta function at 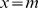, we have 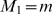. Assuming that in L5 the corresponding activity distribution consists of delta functions at positions 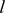, 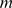, and 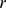, the total input to the perceptron becomes 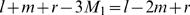. If the bisection stimulus corresponds to the class 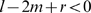, i.e. 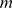 being on the right of the stimulus center, any activity distribution 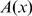 in L2/3 for which 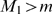 also correctly classifies that stimulus with even a larger margin, i.e. 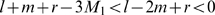. Similarly, for bisection stimulus 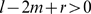, any activity distribution with 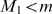 satisfies 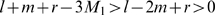. Note that these inequalities remain true if both the activities in L2/3 and L5 are scaled by the same factor.

A way to study how well the network can solve the bisection task therefore consists in showing that, with 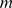 moving to the right, the first order moment grows faster than 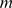, i.e. 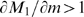 (and vice versa for 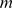 moving left). We show that this is in fact the case, both through simulations and analytical considerations, provided that the recurrent projection width, 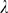 (see Eq. 2), roughly matches half the bisection width.

#### Neuronal field dynamics in V1

Since we are interested in the steady-state activity, we assume that the global recurrent inhibition is fast compared to the temporal dynamics of the activity 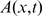 of the excitatory L2/3 neurons. This activity distribution is then governed by a Wilson-Cowan type equation (cf. also [37]),
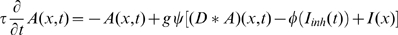
(11)

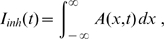
with 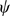 and 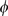 given by Eqs 4 and 7, respectively, and 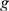 being the neuronal gain factor. The convolution refers to the space variable, 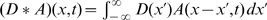, and the kernel of the recurrent weights is given by 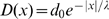, with the same 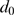 and 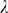 as given after Eq. 2. The input to the L2/3 layer is formed by the sum of the Gaussian projections from the 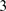 bisection lines, 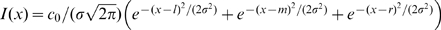, with the same 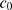 and 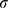 as in Eq. 1.

**Figure 7:**
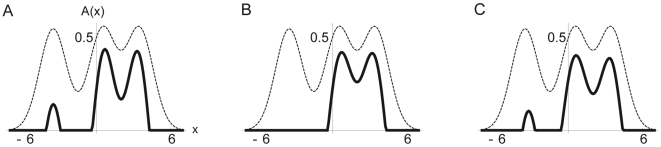
Steady state activity 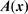 of the continuously distributed L2/3 neurons (Eq. 11, solid lines). Feedforward input 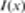 (dashed lines) and bisection stimulus with lines positions at 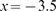, 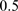 and 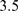, are the same as in Figure 2B. The width of the recurrent projections (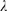) varies for the three sub panels: (A) 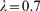, (B) 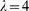 and (C) 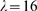. Symmetry breaking is strongest if 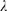 is roughly half the bisection width (B, corresponding to the parameter choice in the discrete simulations). For smaller and larger 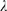 (A and C), the activity to the left bisection line is not fully suppressed and the distribution is less asymmetric (as expressed by a smaller first order moment 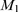, taking on values 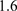, 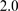 and 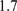 from left to right).

We simulated the neuronal field dynamics (11) with all the parameter values as for the discrete simulations (but without noise), using the slightly asymmetric bisection stimulus shown in Figure 2B with 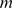 slightly to the right of the stimulus center (here assumed to be at 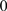, see Figure 7B). According to the simulations, the asymmetry of 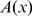 with respect to the bisection center was largest if 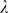 roughly matches half the bisection width 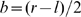, while for smaller and larger projection width the distribution becomes more symmetric (Figure 7). This is also confirmed by evaluating the first order moment 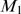 for the different 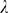
's (see Figure 7 – for comparison we slightly adapted the gain of the inhibitory function to ensure the same overall activity integral). Hence, bisection learning with readout from L5 and L2/3 must be best achievable if 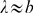. Note, however, that the symmetry breaking property of the recurrent processing is still present (as revealed by the comparison of L2/3 activity with the input in Figure 7), even if 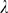 overall changes by a factor of more than 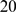 (while keeping the width of the bisection stimulus fixed).

#### Sensitivity analysis for the symmetry breaking

To analytically study the sensitivity of the symmetry breaking as a function of the recurrent projection width we consider the steady state solution 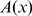 of (11) for transfer functions 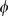 and 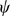 linearized around the region of interest. Introducing the linear operator 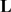 which acts on functions of 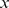, 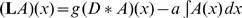, we obtain for the steady state of (11):
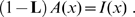
Here, 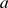 is the slope of the threshold linear inhibitory transfer function and 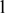 represents the identity function. We invert this equation using the Neumann series – a function operator version of the summation formula for geometric series – and approximate 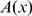 with the zero'th and first order term,
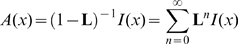


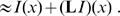
(12)


Next we consider the steady state solution 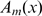 of the full nonlinear equation (11), with index 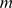 referring to the position of the middle bisection line. Motivated from the above consideration we introduce the *symmetry breaking index* 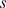 as the derivative of the first order moment 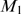 with respect to 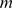, when 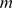 is at the bisection center (assumed to be at 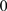). Hence, in recalling its dependency on the recurrent projection width 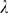, we define
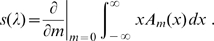



To simplify the calculation we assume delta-like inputs to the L2/3 network generated by the bisection stimuli, 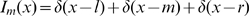. Since the nonlinear inhibition always suppresses the activity slightly outside of the bisection stimulus (cf. Figure 7), we restrict the above integral to the range of the bisection stimulus, from 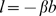 to 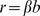. The parameter 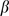 incorporates the effect of the nonlinear suppression, with maximal suppression for 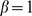 in the case of strong recurrence, and weaker suppression, say 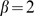, in the case of weaker recurrence. Taking account of the nonlinearities in this way, we plug 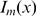 into the linear approximation (12) and obtain for the symmetry breaking index
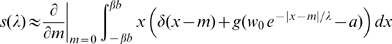
(13)

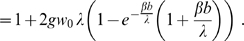
(14)Note that in (13) the terms containing 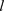 and 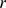 cancel due to the symmetry with respect to the origin, 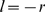, and the integration in the presence of the factor 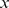. Similarly, the gain of the inhibition, 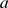, drops out in (14) by symmetry. The first ‘
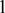
’ in (14) is obtained from the derivative of the integral across the delta term and describes the motion of the line at position 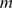 to the right with unit speed. The rest of (14) describes how the movement of the center of gravity with 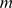 moving to the right is modulated by the lateral interactions.

**Figure 8:**
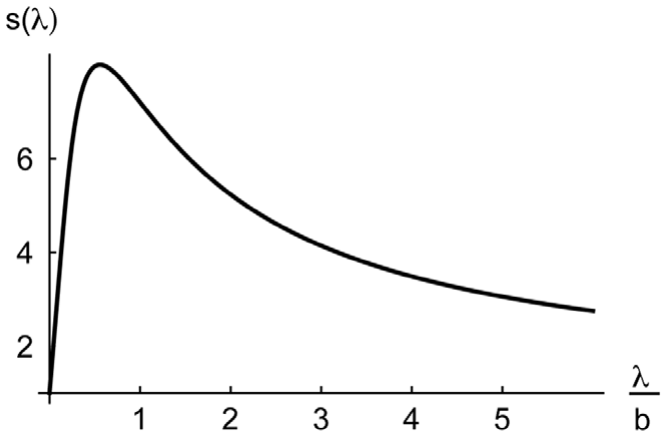
Symmetry breaking as a function of the recurrent projection width (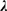). The symmetry breaking index 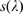 describes the shift in the center of gravity of the L2/3 steady state activity when displacing the middle bisection line away from the bisection center (Eq. 14). Parameter values: 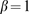, and the same values 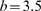, 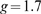, and 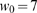 as in the other simulations. The maximum of 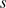 is at 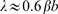, confirming that (for a nonlinear suppression parameter 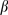 between 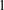 and roughly 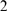) the optimal recurrent projection width (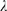) is in the range of the half width of the bisection stimulus (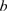).

Numerical evaluation of the function (14) confirms that symmetry breaking is strongest for 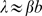 (Figure 8). Further, given a fixed ratio 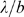, the symmetry breaking index increases with the gain 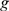 and the recurrent connection strength 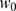 (see Eq. 14). But 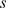 also increases since the suppression parameter 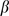 decreases towards 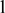 when the competition increases, for instance through an increase of 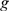, 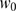, or the gain of the global inhibition, 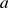. For the more general case where the delta-like input is replaced by a smoothed version with projection width 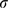, we obtain a similar dependency of 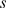 on 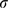.

#### A psychophysical prediction

The recurrent projection width 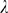 sets an intrinsic scale for the representation of the bisection stimuli. When fixing 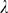 while increasing the half bisection width 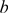, the symmetry breaking index 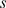 decreases according to Eq. 14. As a consequence, the L2/3 activity is less sensitive to changes of the middle bar position at the stimulus center, and the performance in the bisection task is predicted to decrease. However, when increasing the width of the bisection lines, 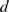, so that the effective projection width, 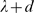, is again in the order of the half stimulus width 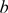, the performance is predicted to be recovered.
